# Multiple Myeloma: Case of a “Moving” Sternal Wire

**DOI:** 10.3390/diagnostics13122082

**Published:** 2023-06-16

**Authors:** Yun Song Choo, Melissa Gaik-Ming Ooi, Shi Wang, James Thomas Patrick Decourcy Hallinan

**Affiliations:** 1 Department of Diagnostic Imaging, National University Hospital, Singapore 119074, Singapore; james_hallinan@nuhs.edu.sg; 2 Division of Haematology, Department of Haematology-Oncology, National University Cancer Institute, Singapore 119074, Singapore; 3 Department of Pathology, National University Hospital, Singapore 119074, Singapore

**Keywords:** multiple myeloma, MRI, PET/CT

## Abstract

Multiple myeloma generally occurs in older adults, with the clonal proliferation of plasma cells and accumulation of monoclonal protein resulting in a broad range of clinical manifestations and complications, including hypercalcemia, renal dysfunction, anaemia, and bone destruction (termed CRAB features). A 64-year-old man with no history of malignancy presented with an enlarging precordial lump occurring three years post-sternotomy for uneventful coronary artery bypass grafting surgery. Initial investigations showed anaemia and impaired renal function. Multimodal imaging performed for further evaluation showcases the radio-pathological features which can be encountered in haematological malignancy. Subsequent percutaneous biopsy confirmed an underlying plasma cell neoplasm, and a diagnosis of multiple myeloma was achieved. The prompt resolution of the lesions upon the initiation of treatment highlights the importance of early diagnosis and treatment.

**Figure 1:**
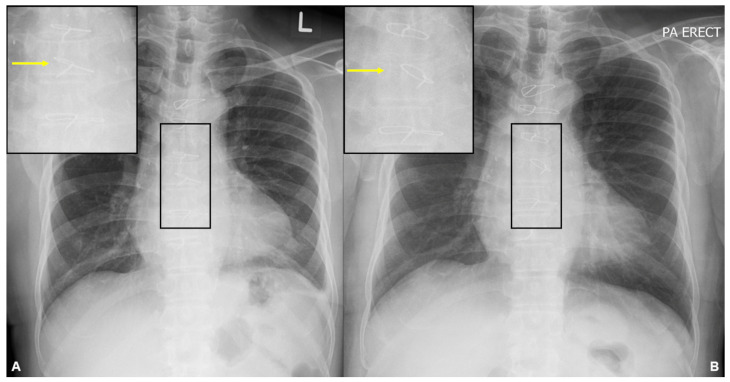
A frontal chest radiograph was obtained on presentation (**B**), showing left lateral displacement of a sternal wire in a chest radiograph from 3 years ago (**A**). No fracture of the sternal wires is seen. Initial laboratory investigations showed a low haemoglobin level of 8.8 g/dL. The platelet and white cell counts were within normal limits. The serum creatinine level was elevated (149 µmol/L; estimated glomerular filtration rate of 42 mL/min) with normal serum calcium and electrolyte levels. The liver function tests were normal, and the serum alkaline phosphatase (ALP) and lactate dehydrogenase (LDH) levels were not elevated.

**Figure 2:**
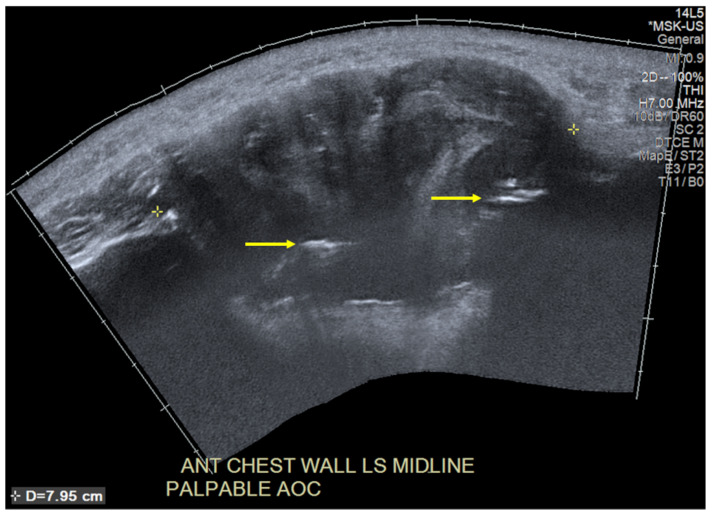
Targeted ultrasonography of the anterior chest wall shows a heterogeneous hypoechoic 7.95 cm mass encasing the sternal wires, which are seen as linear hyperechoic structures indicated by the yellow arrows. The sternum’s haematopoietic marrow and rich blood supply serve as ideal substrates for the development of tumours and infections. The majority of sternal lesions are malignant [1], with metastases being most common in adults.

**Figure 3:**
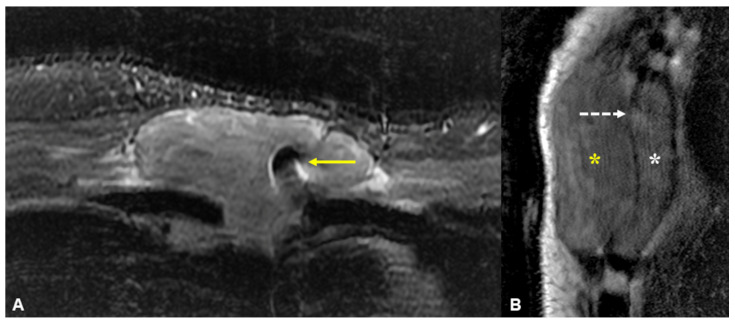
Magnetic resonance imaging (MRI) of the anterior chest wall with selected axial fat-suppressed short-tau inversion recovery (STIR, (**A**)) and sagittal T1-weighted (**B**) images. A fairly homogeneous 8.7 cm mass is seen encasing a sternal wire, appearing as focal susceptibility (yellow arrow). The sagittal image shows diffuse marrow infiltration in the deformed and expanded sternal body (white asterisk) with a large extra-osseous mass extending into the anterior soft tissues (yellow asterisk) through a relatively preserved cortex (linear hypointense signal indicated by the dotted white arrow). While this appearance has been described in osseous lymphoma [2], it can also occur in other malignant round cell lesions of bone, including plasma cell neoplasms [3]. This is likely due to permeative tumour growth via cortical neurovascular channels, allowing relative cortical preservation despite the large extra-osseous component. Other differentials for this MRI appearance include Ewing sarcoma or primitive neuroectodermal tumour (PNET), but these occur predominantly in the paediatric population. Histological evaluation of the lesion revealed a plasma-cell-rich lesion with occasional plasma cells demonstrating nuclear atypia and plasmablastic-type morphology. The plasma cells also show evidence of kappa immunoglobulin light chain restriction using in situ hybridization techniques. Bone marrow biopsy demonstrated an infiltrate of neoplastic plasma cells within the intertrabecular spaces, comprising 60% of the nucleated cell population. Further laboratory investigations revealed elevated total serum protein levels of 105 g/L (normal range 65–82 g/L), with a kappa free light chain (FLC) level of 256.7 mg/L (normal range 3.3–19.4 mg/L) and free kappa-lambda ratio of 42.08 (normal range 0.26–1.65). The IgG level was 53.2 g/L (normal range 5.0–15.0 g/L) and B2-microglobulin level was 5.59 mg/L (normal range 0–1.9 mg/L). Cutaneous plasmacytomas have been known to develop at sites of previous surgery or trauma, whether as a secondary lesion in patients with known plasma cell disorders [4,5] or as a primary lesion [6,7]. However, we believe that this is the first description of a primary plasma cell disorder presenting with both osseous and extramedullary disease at the site of previous sternotomy. The predilection of plasma cell neoplasms for sites of injury or scar formation is not well understood. It has been postulated that the inflammatory mediators present in scar tissue to facilitate wound healing (such as interleukin-6, interleukin-8, and tumour growth factor-β) can result in the selection of a clonal plasma cell population and also support tumour growth and survival [5,7].

**Figure 4:**
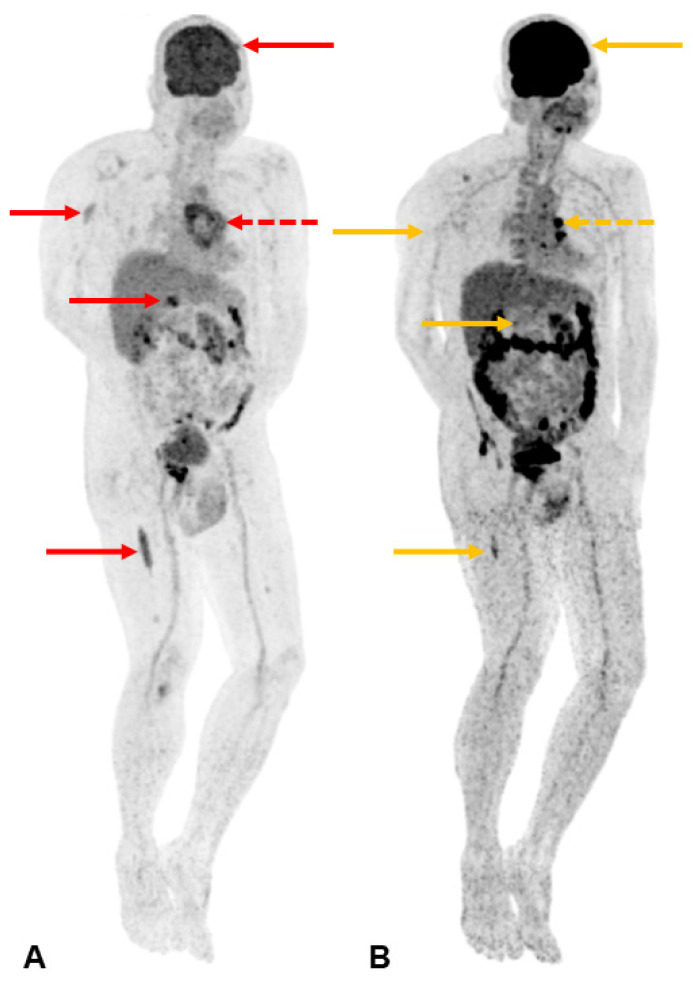
A subsequent 18-fluorodeoxyglucose (FDG) whole-body positron emission tomography-computed tomography (PET-CT) study (**A**) shows intense FDG avidity at multiple sites, including the index sternal lesion (SUVmax 4.9) [indicated by the dotted red arrow] and other foci in the left frontal skull (1.5 cm, SUVmax 4.6), right humerus (4.4 cm, SUVmax 3.5), lower thoracic vertebra (2.1 cm, SUVmax 7.4), and right femur (9.5 cm, SUVmax 4.7) [indicated by solid red arrows in a cephalocaudal direction, respectively]. The patient’s multifocal osteolytic lesions, anaemia with a haemoglobin level less than 10 g/dL, and a bone marrow clonal plasma cell percentage of 60% were consistent with a diagnosis of multiple myeloma, as set out in the 2014 International Myeloma Working Group (IMWG) criterion [8]. Bone disease is present in up to 90% of multiple myeloma patients. It is related to increased osteoclastic activity, mediated by osteoclast-activating factors and local cytokines such as interleukin-6 and tumour necrosis factor-α. This can result in diffuse osteoporosis, solitary or multiple osteolytic lesions, fractures, and hypercalcemia from increased bone turnover. Imaging plays a pivotal role in the detection and assessment of the extent of bone disease. In suspected multiple myeloma, low-dose whole-body CT or FDG PET-CT is recommended to identify osteolytic lesions (>5 mm in size) [9]. Focal osseous radiotracer uptake on FDG PET-CT alone is insufficient to fulfil the IMWG criterion for bone lesions in the absence of osteolysis. If CT or PET-CT is negative or inconclusive, MRI can be a more sensitive modality in detecting focal lesions (>5 mm in size) before osteolytic bone destruction is evident [9]. Radiographic skeletal surveys are no longer recommended as a first-line investigation. In terms of staging and prognostication, the 2005 International Staging System (ISS) score considers two parameters—high serum β2-microglobulin levels reflect a high tumour burden, while low serum albumin levels are related to the presence of inflammatory cytokines such as interleukin-6 in the myeloma microenvironment. The Revised International Staging System (R-ISS) [10] incorporates two additional parameters that demonstrate impact on survival—the elevation of serum LDH level reflects a high proliferation rate, while the presence of high-risk cytogenetic abnormalities (namely, del(17p), translocation t(4;14)(p16;q32), or translocation t(14;16)(q32;q23)) are associated with aggressive biological behaviour. Patients with R-ISS stage I, II, and III have 5-year overall survival rates of 82%, 62%, and 40%, respectively [11]. Prompt treatment of active multiple myeloma is essential to prevent further complications and organ damage. The addition of autologous stem cell transplantation has been associated with longer progression-free survival [12], while novel agents such as daratumumab have also increased treatment efficacy [13]. Our patient was able to achieve response with a regimen of daratumumab, bortezomib, thalidomide, and dexamethasone (D-VTd). The precordial lump was no longer palpable after the first cycle of systemic therapy in the repeat FDG PET-CT study conducted three months after the initiation of treatment, showing a marked improvement in the previously seen lesions (**B**). Local radiotherapy to the index sternal lesion was not offered to the patient due to the prompt response to systemic therapy with resolution of the pain associated with the precordial lump. The other osseous lesions were also not symptomatic, negating the need for palliative radiotherapy [14]. Six months after the initial diagnosis, our patient also underwent autologous stem cell transplant which was shown to improve progression-free survival in the CASSIOPEIA trial [15].

## Data Availability

Not applicable.
